# Genome-wide DNA methylome analysis reveals a critical role of methylation-dysregulated lncRNAs in autophagy regulation in glioblastoma

**DOI:** 10.1016/j.gendis.2023.101107

**Published:** 2023-09-12

**Authors:** Hongying Zhao, Ying Liu, Meiting Fei, Lin Bo, Lixia Wang, Yaopeng Shu, Peiqi Ben, Li Wang

**Affiliations:** College of Bioinformatics Science and Technology, Harbin Medical University, Harbin, Heilongjiang 150081, China

Glioblastoma (GBM) is the most common primary malignant intracranial tumor with a very poor prognosis. In this study, we systematically analyzed the DNA methylation alterations of autophagy-related lncRNAs and their association with drug therapies in GBM. We identified 9 DNA methylation-dysregulated lncRNA regulators of autophagy-related genes as autophagy-related lncRNAs. A dysregulated regulatory network consisting of 9 autophagy-related lncRNAs and 237 differentially expressed autophagy-related genes was constructed. Identification of small molecule drug candidates that may affect DNA methylation dysregulated lncRNA activity, including 45 drug lncRNA pairs, involving 7 DNA methylation dysregulated lncRNAs and 43 drugs. Furthermore, we identified a DNA methylation-dysregulated autophagy-related lncRNA MIR155HG as an independent prognostic indicator for GBM. The significantly decreased methylation level of the MIR155HG promoter contributed to its up-regulated expression, which was involved in autophagy regulation through the regulation of autophagy-related genes *WMP1*, *AP4M1*, and *UBQLN2*. Our results showed that DNA methylation-dysregulated lncRNAs may be potential biomarkers and drug targets in GBM through regulating autophagy-related functions.

We first performed differential expression analysis of lncRNAs based on RNA-seq data from TCGA to obtain 3080 differentially expressed lncRNAs (Fig. 1A, B; Fig. S1A). We performed differential DNA methylation analysis between 141 Infinium Human Methylation 450K assay GBM tumor tissues and 50 normal tissues using the LncDM R package. We identified 171 differentially methylated sites with a false discovery rate <0.05 and 142 lncRNAs with differentially methylated sites in the promoter regions. There are multiple sites of differential methylation in PVT1 (Fig. S1B). We also characterized abnormally methylated sites in the enhancer region using the ChAMP package based on Illumina 850k EPIC array data from the 112 glioma and 38 normal brain samples. A total of 59 DNA methylation-dysregulated lncRNAs were identified when at least one differential methylation site was located in the promoter/enhancer regions of differentially expressed lncRNAs and exhibited opposite correlations between DNA methylation and lncRNA expression^1^^,^^2^ (Table S1 and Fig. S1C), including 15 differentially methylated lncRNAs at promoter regions and 48 differentially methylated lncRNAs at enhancer regions.

**Figure 1:**
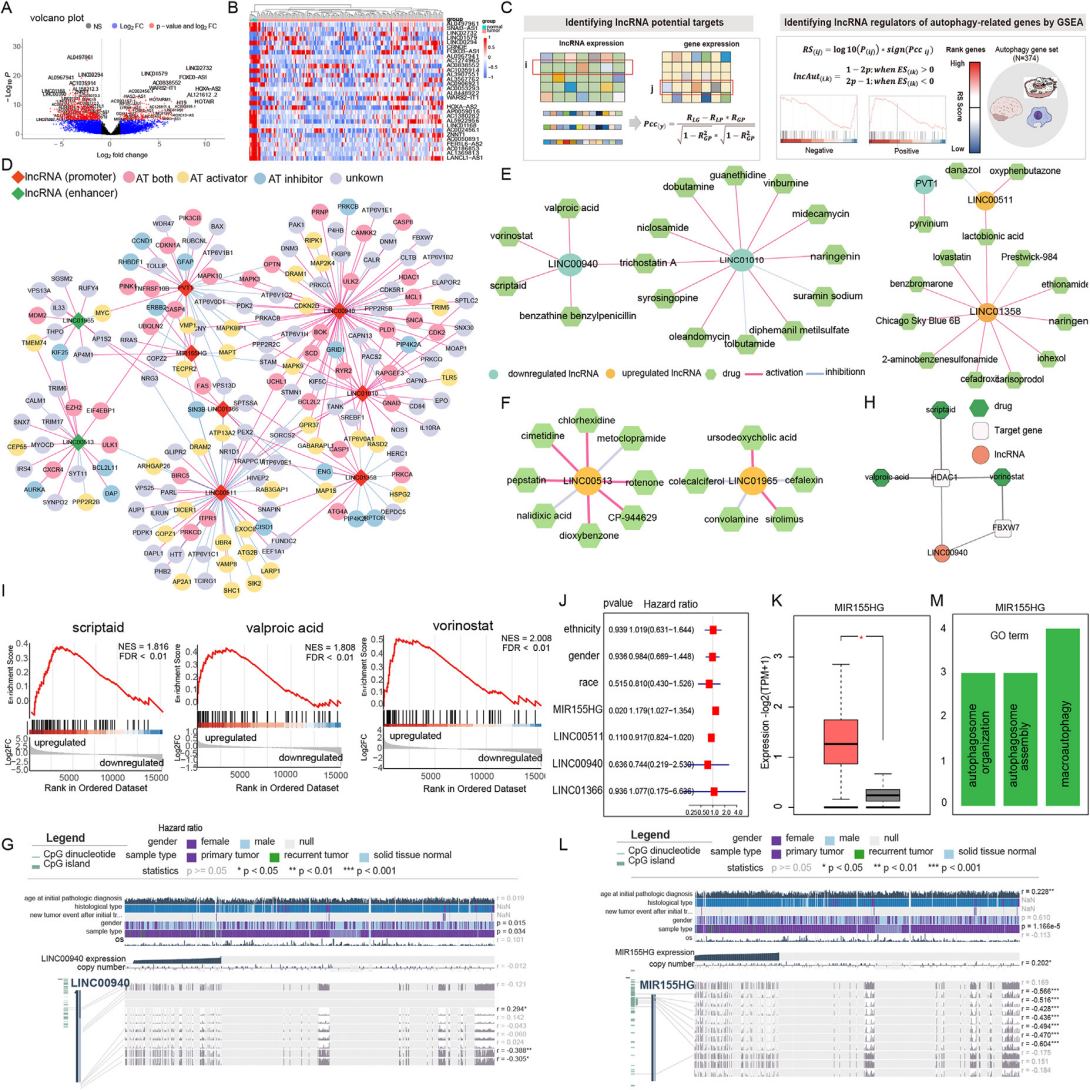
Genome-wide DNA methylome analysis reveals a critical role of methylation-dysregulated lncRNAs in autophagy regulation in glioblastoma. **(A)** Volcano plot showing up- and down-regulated lncRNAs. **(B)** Expression profile of the top 30 differentially expressed lncRNAs between tumors and normal tissues. The color scale represents the relative expression level for each lncRNA. **(C)** Three-step algorithm based on GSEA enrichment analysis. **(D)** Co-expressed network of DNA methylation-dysregulated autophagy-related lncRNAs and autophagy-related genes. The red and green diamonds represent autophagy-related lncRNAs with DNA methylation dysregulation in promoter regions and enhancer regions, respectively. The circles represent autophagy-related genes. The fill colors of the circle nodes indicate the activators (yellow), inhibitors (blue), both an activator and a suppressor (pink), and unannotated (gray) of autophagy-related genes according to the THANA TOS database. The red edges represent the positive (red) or negative (blue) regulation of lncRNA on autophagy-related genes. **(E)** Drug-lncRNA network was constructed, involving 5 autophagy lncRNAs with differentially methylated promoters and 30 drugs. **(F)** Drug-lncRNA network, involving 2 autophagy lncRNAs with differentially methylated enhancers and 13 drugs. **(G)** The DNA methylation of LINC00940 visualized by the MEXPRESS website (https://mexpress.be/). **(H)** Visualization of the regulatory network constructed by LINC00940, three small molecule compounds, and two target genes. **(I)** GSEA results for LINC00940 and 3 drug candidates. The bottom panel shows the differential expression profiles of small molecules for each drug candidate; the top panel shows the continuous enrichment scores of the drug candidates, indicating whether the gene set is enriched in up-regulated or down-regulated regions. **(J)** The forest plot presenting the independent prognostic analysis for MIR155HG. **(K)** The expression levels of MIR155HG between tumor and normal tissues were verified in the GEPIA database. The database includes 207 normal samples and 163 GBM samples. The red box on the left and the gray on the right are the tumor group and normal group respectively. **(L)** The DNA methylation of MIR155HG visualized by the MEXPRESS website (https://mexpress.be/). **(M)** GO functional enrichment of MIR155HG target genes.

To identify autophagy-related candidate lncRNA regulators, we used a three-step computational algorithm based on GSEA functional enrichment (Fig. 1C). The core idea of the algorithm is that when genes co-expressed with lncRNA are enriched in the autophagy gene set, and then we reasoned that the lncRNA will also play an important role in autophagy. First, a total of 374 differential autophagy genes were obtained from the intersection between GBM differential genes (Fig. S2A) and autophagy database genes. Second, we calculated the tumor purity of each patient and ranked the genes according to the RS score of each candidate lncRNA. Third, we calculated the score of lncRNA among differential autophagy genes (lncAut) based on gene set enrichment analysis (GSEA). The *P*-value of GSEA was converted to a lncAut score and lncRNAs with lncAut > 0.9 and a false discovery rate < 0.05 were selected. Finally, 9 autophagy-related lncRNAs in GBM were obtained (including up-regulation of LINC00511, LINC01358, LINC01366, MIR155HG, PVT1, LINC01965, and LINC00513 and down-regulation of LINC00940 and LINC01010) (Table S2 and Fig. S2B, C).

In order to further analyze the relationship between DNA methylation-dysregulated autophagy-related lncRNAs and 374 differentially expressed autophagy-related genes, we constructed a dysregulation regulatory network. The dysregulated regulatory network consisted of 9 autophagy-related lncRNAs and 237 differentially expressed autophagy-related genes (Fig. 1D). The 374 autophagy-related genes are divided into four groups, including promoting autophagy (AT+), inhibiting autophagy (AT−), promoting and inhibiting (AT both) autophagy, and unclassified by THANATOS. The significantly increased DNA methylation level of hub lncRNA LINC00940 contributed to its down-regulated expression in cancer patients. LINC00940 showed a significantly positive correlation with autophagy-promoting genes (*P* = 5.6e-3), and a significantly negative correlation with autophagy-inhibiting genes (*P* = 2.8e-3), indicating a potential role of LINC00940 promoting autophagy (Fig. S3). MIR155HG positively regulates the autophagy inhibitor ERBB2, and ERBB2 plays an important role in the PI3K−Akt signaling pathway (Fig. S4). Studies have found that ERBB2 is a therapeutic target for glioma, and it happens to affect glioma through the PI3K−Akt signaling pathway.^3^ In conclusion, our research underlined that DNA methylation-dysregulated lncRNAs could regulate the expression of autophagy genes, thereby affecting autophagy-related pathways, and ultimately playing a role in the occurrence and development of GBM.

As non-coding RNAs, lncRNAs may affect the expression of cancer-related genes and may represent a class of potential targets for drug discovery. We generated an integrative pipeline to identify candidate small molecules that can affect autophagy-related lncRNA activity. First, we obtained co-expressed autophagy genes of 9 autophagy-related lncRNAs as their potential target genes. Next, based on the gene set enrichment analysis (GSEA) method, we calculated whether the target genes of autophagy-related lncRNAs are significantly affected by drug perturbation based on the Broad Institute's Connectivity Map (CMap). Based on false discovery rate < 0.01, a total of 45 candidate drug-lncRNA relationship pairs (Table S3) were identified, involving 7 autophagy lncRNAs and 43 drugs (37 pairs of drug-lncRNA NES > 0; 8 pairs NES values < 0) (Fig. 1E, F). For example, LINC00940 is significantly down-regulated in GBM tissues that could be affected by DNA methylation (Fig. 1G). We identified 3 candidate drugs that were predicted to induce LINC00940 expression, including scriptaid, valproic acid, and vorinostat (Table S4 and Fig. 1H). We found that LINC00940 co-expressed autophagy gene HDAC1 is the target gene of scriptaid, valproic acid, and vorinostat. HDAC inhibitor vorinostat is a drug candidate that promotes LINC00940 expression by affecting the known target genes FBXW7 and HDAC1 (Fig. S5A). HDAC inhibitor vorinostat is a drug candidate that promotes LINC00940 expression by affecting the known target genes FBXW7 and HDAC1. According to the results of GSEA enrichment analysis, co-expressed autophagy genes of LINC00940 tend to be up-regulated under drug perturbation (Fig. 1I). In summary, we identified drug candidates for autophagy-related lncRNAs, which may disrupt lncRNA expression by targeting autophagy-related genes and could be considered as potential anticancer drug targets.

To gain insights into the potential prognostic value of DNA methylation-dysregulated autophagy-related lncRNA, we analyzed the expression of 9 autophagy-related lncRNAs. Four lncRNAs significantly associated with survival prognosis were obtained by Kaplan–Meier curve and log-rank test (Fig. S5B). Furthermore, we performed a multivariate Cox proportional hazards model analysis on the expression of these 4 autophagy-related lncRNAs in relation to clinical parameters such as gender, race, and ethnicity. We identified one lncRNA MIR155HG with promoter hypomethylation and up-regulated expression as independent risk factors for GBM prognosis (Fig. 1J–L). Functional analysis showed that MIR155HG was involved in autophagosome organization, autophagosome assembly, and macroautophagy through targeted regulation of autophagy-related genes WMP1, AP4M1, and UBQLN2 (Table S5 and Fig. 1M). MIR155HG was reported to be up-regulated and a potential biomarker for prognosis and an immunotherapeutic target in glioma.^4^ MIR155HG expression showed a significantly positive correlation with autophagy-related genes UBQLN2 and AP4M1, negatively correlated with autophagy activator WMP1 in GBM, showing the important role of MIR155HG in regulating autophagy in GBM. MIR155HG has been shown to be an autophagy-related lncRNA and has an important prognostic value in acute myeloid leukemia.^5^ Collectively, our findings underline the crucial roles of DNA methylation-dysregulated autophagy-related lncRNAs in breast cancer carcinogenesis and their potential prognostic value and may provide a promising tool for improving the development of GBM risk stratification and personalized treatment.

In summary, our research has revealed the key role of aberrant methylation of lncRNA in the regulation of autophagy, which can become a potential biomarker and drug target related to autophagy in GBM. Our research fills the gap in exploring the regulatory functions and mechanisms of epigenetic modification of autophagy in cancer, as well as in the development of drugs targeting epigenetic and autophagy, and has discovered the signaling pathways for epigenetic modification of autophagy.

## Author contributions

HYZ, YL, and LW designed the study, implemented the algorithm, and performed the analysis. HYZ, YL, LW, MTF, LB, LXW, YPS, and PQB wrote and revised the manuscript. All authors read, reviewed, and approved the final manuscript.

## Conflict of interests

The authors have no relevant financial or non-financial interests to disclose.

## Funding

This work was supported by the University Nursing Program for Young Scholars with Creative Talents in Heilongjiang Province, China (No. UNPYSCT-2020174), the Excellent Youth Project of Provincial Scientific Research Institute (China) (No. CZKYF2022-1-C006), and the Hei Long Jiang Postdoctoral Special Foundation (China) (No. LBH-TZ1018).

## Data availability

All data and codes in this study are available under proper request, please contact wangli@hrbmu.edu.cn.
