# Supplementary material for: Genome-wide DNA methylome analysis reveals a critical role of methylation-dysregulated lncRNAs in autophagy regulation in glioblastoma
Source: Genes Dis. 2023 Sep 12;11(5):101107. doi: 10.1016/j.gendis.2023.101107 (PMC11167244; doi:10.1016/j.gendis.2023.101107)
Supplement: Multimedia component 1 [file mmc1.pdf]

Supplementary Information for

Genome-wide DNA methylome analysis reveals a critical role of methylation-dysregulated lncRNAs in autophagy regulation in glioblastoma

Hongying Zhao[1,$], Ying Liu[1,$], Meiting Fei[1], Lin Bo[1], Lixia Wang[1], Yaopeng Shu[1], Peiqi Ben[1], Li Wang[1,*]

1College of Bioinformatics Science and Technology, Harbin Medical University, Harbin 150081, China.

$These authors contributed equally to this work.

***Corresponding Authors:**

Li Wang, College of Bioinformatics Science and Technology, Harbin Medical University, Harbin 150081, China. Tel.: 86-451-86615922, E-mail: wangli@hrbmu.edu.cn

This PDF file includes:

SI Materials and Methods

SI Results

SI Figures and Tables

SI Material and Methods

Datasets

The DNA methylation array data (Illumina Infinium Human Methylation 450 BeadChip), and RNA-sequencing dataand clinicopathological information of TCGA GBM were downloaded from the UCSC Xena browser. Autophagy-related genes were obtained from several autophagy-related databases, including HADB, HAMdb, THANATOS, ATD and AmiGO 2. The drug-protein intersections were downloaded from DrugBank and the drug-gene interaction database (DGIdb). In order to explore the relationship between the drug and its target, we obtained more than 7000 expression profile from the CMap database for 1309 compounds and vector controls experiments. For each compound, the differential expression profiles were calculated as the mean level of difference between drug and vector controls across all experiments and cell lines, then we generated a ranked gene lists by sorting the differential gene expression profiles from the most up-regulated to the most down-regulated genes.

Identification of differentialy expressed genes and lncRNAs

The R package DEseq2 was used to analyze the difference in gene expression values between TCGA 5 normal and 689 GBM in the mRNA expression profile and lncRNA expression profile, and finally obtain differential genes and differential lncRNAs. The threshold values were $|\log2\ FC| > 1$ and $P<0.05$. The results of the analysis can be visualized by drawing heatmaps and volcano maps with the R package heatmap and ggplot2. We also identified 1567 differentially expressed lncRNAs by comparing TCGA GBM tumors versus 53 normal brain tissues from both TCGA and Genotype-Tissue Expression (GTEx), which are significantly consistent with those identified in TCGA data (*P*-value=6.26e-72; hypergeometric test).

Identification of differentially methylated lncRNAs

We identified the regulatory relationship between long non-coding RNA (lncRNA) and DNA methylation in GBM, and searched for differentially methylated sites. We performed differential methylation analysis using R package LncDM (http://lncdm.r-forge.r-project.org/) and identified 171 differentially methylated lncRNAs in promoter regions. In order to identify lncRNAs affected by aberrantly methylated sites in enhancer regions, the 850K methylation microarray including 112 glioma samples (GSE188547) and 38 normal brain tissue samples (GSE195834) were obtained from the GEO database. The acquired methylated sites were annotated based on the 850K platform annotation information. After missing values were imputed by the impute.knn function in the impute package of ChAMP was used for subsequent analysis. Firstly, correct the probe to eliminate the error caused by the technical difference of the two types of probes by using the champ.norm function[1]. The 164,467 differentially methylated CpG sites (DMGs) were selected with significant cut-off values of false discovery rate (FDR) <0.05 and |log fold change (logFC)| > 0.2[2]. The ATAC-seq peak accessibility of 404 GBM patients was obtained from the UCSC Xena Browser (https://atacseq.xenahubs.net). The 15,395 ATAC-seq peaks were identified as potential enhancers in GBM. We implemented a strategy based on the correlation of ATAC-seq accessibility and lncRNA expression across GBM samples and restricted the length scale of this analysis to 500 kbp[3]. Using an FDR cutoff of 0.01, we identified the

links between enhancers and lncRNAs. A total of 48 lncRNAs with differentially methylated enhancers were identified when at least one differential methylation site was located in the enhancer regions of differentially expressed lncRNAs and exhibit inversely correlated with lncRNA expression[4;5].

Identification of candidate lncRNA regulators of autophagy in GBM

A three-step computational algorithm based on GSEA enrichment identifies autophagy-related lncRNAs. Identification of lncRNA-correlated genes. In order to avoid the error caused by directly calculating the correlation coefficient, we introduced tumor purity as a covariate to calculate the partial correlation coefficient (PCC) between lncRNAs and genes. Define lncRNA, gene expression value, and sample tumor purity as L, G, and P respectively. R_{LG}, R_{LP} and R_{GP} represent the correlation coefficient between lncRNA and gene, lncRNA expression value and tumor purity, gene expression value and tumor purity, respectively as follow:

$$PCC(ij) = \frac{R_{LG} - R_{LP} * R_{GP}}{\sqrt{1 - R_{LP}^2} * \sqrt{1 - R_{GP}^2}}$$

Then, we compute the rank score (RS) for each pair of PCC values:

$$RS(ij) = -log10\big(p(ij)\big) * sign(PCC(ij))$$

Genes were ranked based on RS scores, followed by GSEA enrichment analysis. Finally, we combined the ES score and p-value obtained by GSEA enrichment into lncAut score, i.e.,

$$lncAut(i,k) = \begin{cases} 1 - 2p \; ; \; ifES(ik) > 0, \\ 2p - 1 \; ; \; ifES(ik) < 0. \end{cases}$$

We consider the lncRNAs in the relationship between lncAut>0.9 and FDR<0.05 to be autophagy-related lncRNAs.

Survival prognosis of autophagy-related lncRNAs

By Kaplan-Meier survival analysis, the log-rank test method was used to compare the differences in OS results between high and low expression lncRNAs. In order to characterize whether the survival-related lncRNA has independent prognostic ability, we constructed a multi-factor cox regression model, and finally used the forestplot package to draw a forest plot to visualize the results.

Co-expression network construction of abnormally methylated lncRNA and autophagy genes

We used co-expression analysis to identify autophagy target genes of aberrantly methylated lncRNAs. For each screened autophagy-related lncRNA, the expression correlation between each lncRNA and differentially expressed genes was calculated using Spearman rank correlation, and the FDR-BH-adjusted P value was calculated. Consider the genes with FDR<0.001 as the targets of lncRNA, and finally get the direct target gene set of the target lncRNA.

Identification of drug candidates affecting DNA methylation-regulated autophagy-associated lncRNAs

To identify candidate small molecules that affect DNA methylation regulation of autophagy-associated lncRNAs, we first identified the set of co-expressed target genes

of autophagy-associated lncRNAs, and generated ranked gene lists from drug-induced gene expression profiles based on the Broad Institute's Connectivity Map. We then calculated whether drug perturbations would significantly affect the target genes of autophagy-related lncRNAs by performing GSEA on each drug in the Connectivity Map. We considered a predicted drug to affect lncRNA activity if the FDR-adjusted p-value was less than 5% (FDR<0.05) and the normalized enrichment score was used as a score indicating the strength of the prediction. Finally, we calculated the shortest path length between all drug-lncRNA pairs in the biological network.

SI Results

Identification of DNA methylation-dysregulated lncRNAs in GBM

A total of 3080 differentially expressed lncRNAs (1136 up-regulated lncRNAs and 1944 down-regulated lncRNAs) were identified. For example, lncRNA *HOXA-AS2* was significantly upregulated in GBM samples (Figure S1). *HOXA-AS2* expression was proved to promote proliferation of glioma stem cells and modulate their inflammation pathway. LncRNA *SNAI3-AS1* was significantly downregulated in GBM samples. The expression of *SNAI3-AS1* were negatively correlated with glioma malignancy. The high expression of *SNAI3-AS1* may suppress gliomas progression by activating NK cells. A total of 59 DNA methylation-dysregulated lncRNAs were identified when at least one differential methylation site was located in the promoter/enhancer regions of differentially expressed lncRNAs and exhibit opposite correlations between DNA methylation and lncRNA expression. For example, the expression levels of lncRNA *LINC00511* (Pearson correlation coefficient R=-0.30, P=0.02), *LINC01358* (R=-0.33, P=6.9e-03) and *PVT1* (R=-0.62, P=3.7e-08) are significantly negatively correlated with these differentially methylated sites. Ten CpG sites in the *PVT1* promoter (cg12480416, cg03457528, cg08334153, cg26900458, cg13784855, cg25247520, cg11201447, cg00780520, cg22853542 and cg23898497) were significantly correlated with *PVT1* expression via MEXPRESS (correlation coefficients from -0.279 to -0.700; Figure S1).

Identification of autophagy-related lncRNAs in GBM

To identify autophagy-related candidate lncRNA regulators, we used a three-step computational algorithm based on GSEA functional enrichment. A total of 9 autophagy-related lncRNAs in GBM are obtained (including upregulation of LINC00511, LINC01358, LINC01366, MIR155HG, PVT1, LINC01965, LINC00513 and downregulation of LINC00940, LINC01010) (Table S2). LncRNA *LINC00511*, *LINC01358* and *PVT1* are significantly highly expressed in GBM, among which *LINC01366* and *LINC00511* were regulated by DNA methylation (Figure S2). Studies have found that *LINC00511* can not only inhibit autophagy and enhance the apoptosis of trophoblast cells, but also target *HOXA7* as a sponge of *miR-31-5p* and indirectly affect autophagy. Long non-coding RNA *PVT1* has been shown to be a potential biomarker for early detection and prognosis prediction of glioma. In the study of intense angiogenesis in glioma, it was found that *PVT1* can increase the expression of autophagy-related proteins *Atg7* and *Beclin1* by targeting *miR-186* (*miR-186* targets the 1' untranslated regions of *Atg7* and *Beclin1* to reduce their expression levels) to induce protective autophagy, thereby promoting the proliferation, migration and angiogenesis of glioma vascular endothelial cells. In the review on the effect of long non-coding

RNA on glioma, it was clearly shown that the oncogenic lncRNA *PVT1* participates in cell apoptosis and autophagy, thereby affecting the formation of glioma. In the review exploring lncRNAs related to autophagy regulation in nervous system diseases, *PVT1* activates autophagy to promote glioma proliferation, migration and angiogenesis. All the results show that the algorithm can identify the key lncRNAs related to glioma autophagy, and these lncRNAs as autophagy-related lncRNAs can be used as a reference for future research on glioma autophagy.

The regulatory network between DNA methylation-dysregulated lncRNAs and autophagy-related genes

The dysregulated regulatory network consisted 9 autophagy-related lncRNAs and 237 differentially expressed autophagy-related genes was constructed. The network diagram showed that *LINC00940* is a hub lncRNA regulating 12 AT activitors, 3 inhibitors and 19 AT_both genes. The significantly increased DNA methylation level of *LINC00940* contributed to its down-regulated expression in cancer patients. *LIN00940* negatively regulates the target gene *DRAM1* which can promote autophagy. The KEGG functional pathway showed that AT activitors (such as *DRAM1*) was significantly enriched on the MAPK signaling pathway (Figure S4). The *DRAM1* encodes the key regulator of autophagy p62, and the expression of *DRAM1* plays a key role in the activation of MAPK pathway and p62-mediated autophagy, providing new clues for the role of autophagy in GBM. We have reason to believe that DNA methylation-dysregulated autophagy-related *LINC00940*, affects the MAPK pathway by regulating the key autophagy factor *DRAM1*, and ultimately regulates GBM energy metabolism and cancer cell migration/ invasion. *LINC00940* also positively regulates the target gene *ATP6V0A1*, which plays an important role in mediating autophagosome-lysosome fusion. The autophagy-related lncRNA *MIR155HG* showed hypomethylation and up-regulated expression. *MIR155HG* positively regulates the autophagy inhibitor *ERBB2* in the network, and *ERBB2* plays an important role in PI3K−Akt signaling pathway. Studies have found that the *ERBB2* gene, as a member of the HER superfamily of receptor tyrosine kinases, is a therapeutic target for glioma, and it happens to affect glioma through the PI3K−Akt signaling pathway. In a study exploring the contribution of IC-lncRNA (immune synergistic lncRNA) to cancer development, *MIR155HG* exists as a central hub of the IC-lncRNA biological network and plays an oncogenic role in various cancers, while the central target gene *ERBB2* is more likely to be druggable and may become a potential target for anticancer drugs. According to the regulatory network, abnormally methylated *PVT1* positively regulates the target gene *MYC*. Studies have found that *MYC*, as a key gene in the regulation of autophagy, is also a target of the Wnt signaling pathway, and its expression also depends on signaling pathways such as PI3K/mTOR. It has been reported that lncRNA *PVT1*, as a regulator of key oncogenic pathway transcription, is an enhancer of MYC and aberrant methylation of *PVT1* also leads to changes in *MYC* expression. GO enrichment and Kyoto Encyclopedia of Genes and Genomes (KEGG) pathway enrichment showed that autophagy-related lncRNAs were significantly associated with regulation of autophagy, the ErbB signaling pathway and MAPK signaling pathway. Through network and functional enrichment, it can be seen that lncRNA regulated by epigenetic modification

DNA methylation.In conclusion, our research underlined that DNA methylation-dysregulated lncRNAs could regulate the expression of autophagy genes, thereby affecting autophagy-related pathways, and ultimately plays a role in the occurrence and development of GBM.

Identifying candidate small molecules affecting the activity of DNA methylation-dysregulated autophagy-related lncRNAs

As non-coding RNAs, lncRNAs may affect the expression of cancer-related genes and may represent a class of potential targets for drug discovery. We generated an integrative pipeline to identity candidate small molecules which can affect autophagy-related lncRNA activity. Based on FDR < 0.01, a total of 45 candidate drug-lncRNA relationship pairs (Table S3) were identified, involving 7 autophagy lncRNAs and 43 drugs (37 pairs of drug-lncRNA NES > 0; 8 pairs NES values < 0). For example, *LINC00940* is significantly downregulated in GBM tissues that could be affected by DNA methylation. We identified 5 candidate drugs which were predicted to induce *LINC00940* expression, including scriptaid (NES=1.82), valproic acid (NES=1.81), vorinostat (NES=2.01), trichostatin A (NES=1.93), and benzathine benzylpenicillin (NES=1.65). We found that *LINC00940* co-expressed autophagy gene *HDAC1* is the target gene of scriptaid, valproic acid and vorinostat. *HDAC* inhibitor vorinostat is a drug candidate that promotes *LINC00940* expression by affecting the known target genes *FBXW7* and *HDAC1* (Figure S5A). The co-expressed gene *HDAC1* is a negatively correlated gene of *LINC00940* and its expression is significantly upregulated in glioma. According to the results of GSEA enrichment analysis, co-expressed autophagy genes of *LINC00940* tend to be upregulated under drug perturbation. It has been shown that scriptaid induces apoptosis and reduces glioma cell proliferation by regulating JNK activation, and reduces telomerase activity in a JNK-independent manner. It has been reported that *HDAC1* could increase the phosphorylated JNK and subsequent activation of the JNK pathway. The *HDAC* family has clinical significance for glioma, in which *HDAC1* is not only a prognostic and immune infiltration indicator, but also a core component of *HDAC1*-related signatures for precise prediction of glioma prognosis. The co-expressed gene *FBXW7* is a positively correlated gene of *LINC00940* and its expression is significantly downregulated in glioma. Vorinostat combined with melatonin activated apoptotic signaling in GBM cells, suggesting that co-treatment of vorinostat and melatonin might be an effective therapeutic strategy for glioma. Existing data have shown that *FBXW7* was downregulated in glioblastoma cancer tissues, and demonstrated the important function of *FBXW7* in promoting glioblastoma apoptosis. In summary, we identified drug candidates for autophagy-related lncRNAs, which may disrupt lncRNA expression by targeting autophagy-related genes, and could be considered as potential anticancer drug targets.

DNA methylation-dysregulated autophagy-related lncRNAs acting as prognostic markers of glioblastoma

To gain insights into the potential prognostic value of DNA methylation-dysregulated autophagy-related lncRNA, we analyzed 9 autophagy-related lncRNA expression in 167 GBM patients. The Kaplan-Meier curve and log-rank test revealed that 4 autophagy-related lncRNA expression were able to significantly distinguish patients in

high-risk groups from those in low-risk groups in terms of overall survival, including *LINC00511* and *LINC00940*, *LINC01366*, and *MIR155HG* (log-rank test P<0.05; Figure S5). The high expression of *LINC01366* and *MIR155HG* had shorter survival than the low-risk patients (log-rank test P=9.8e-03 and P=5.6e-03, respectively). A low expression of *LINC00511* and *LINC00940* was associated with a worse overall survival of GBM patients (log-rank test P=0.01 and P=0.02, respectively). Furthermore, we performed multivariate Cox proportional hazards model analysis on the expression of these 4 autophagy-related lncRNAs in relation to clinical parameters such as gender, race and ethnicity. We identified one DNA methylation-dysregulated autophagy-related lncRNA *MIR155HG* (HR=1.2, 95% CI: 1.05 to 1.37, P=8.0e-03) as independent risk factors for GBM prognosis. It was proved that *MIR155HG* expression was negatively correlated with methylation level of *MIR155HG* promoter region (Figure 1). We observed a significantly decreased methylation level of *MIR155HG* promoter, which contributed to its up-regulated expression. Functional analysis showed that *MIR155HG* was involved in autophagosome organization, autophagosome assembly and macroautophagy through targeted regulation of autophagy-related genes *WMP1*, *AP4M1* and *UBQLN2*. Increasing evidence suggests that induction of lethal macroautophagy/autophagy carries potential significance for the treatment of GBM. These results are consistent with those of previous studies where promoter methylation levels of *MIR155HG* in GBM were remarkably decreased compared with those in lower-grade glioma (LGG). *MIR155HG* was reported to be upregulated and a potential biomarker for prognosis and an immunotherapeutic target in glioma. *MIR155HG* expression showed significantly positively correlated with autophagy-related gene *UBQLN2* and *AP4M1*, negatively correlated with autophagy activator *WMP1* in GBM, showing the important role of *MIR155HG* in regulating autophagy in GBM. *MIR155HG* has been shown to be an autophagy-related lncRNA and has an important prognostic value in acute myeloid leukemia. *AP4M1* is reported to be upregulated and redistributed to neuronal axons under oxygen-glucose deprivation stress and are involved in Alzheimer's disease, neurodegeneration with brain iron accumulation. *AP4M1* gene was significantly associated with overall survival in patients with mesenchymal glioblastoma multiforme. Inactivation of *UBQLN2* expression in HeLa cells reduced autophagic flux and autophagosome acidification. *UBQLN2* was reported to be positively correlated with *UBQLN4* and the increase of *UBQLN4* in neuroblastoma decreased survival. Collectively, our findings underline the crucial roles of DNA methylation-dysregulated autophagy-related lncRNAs in breast cancer carcinogenesis and their potential prognostic value, and may provide a promising tool for improving the development of GBM risk stratification and personalized treatment.

SI Figures and Tables

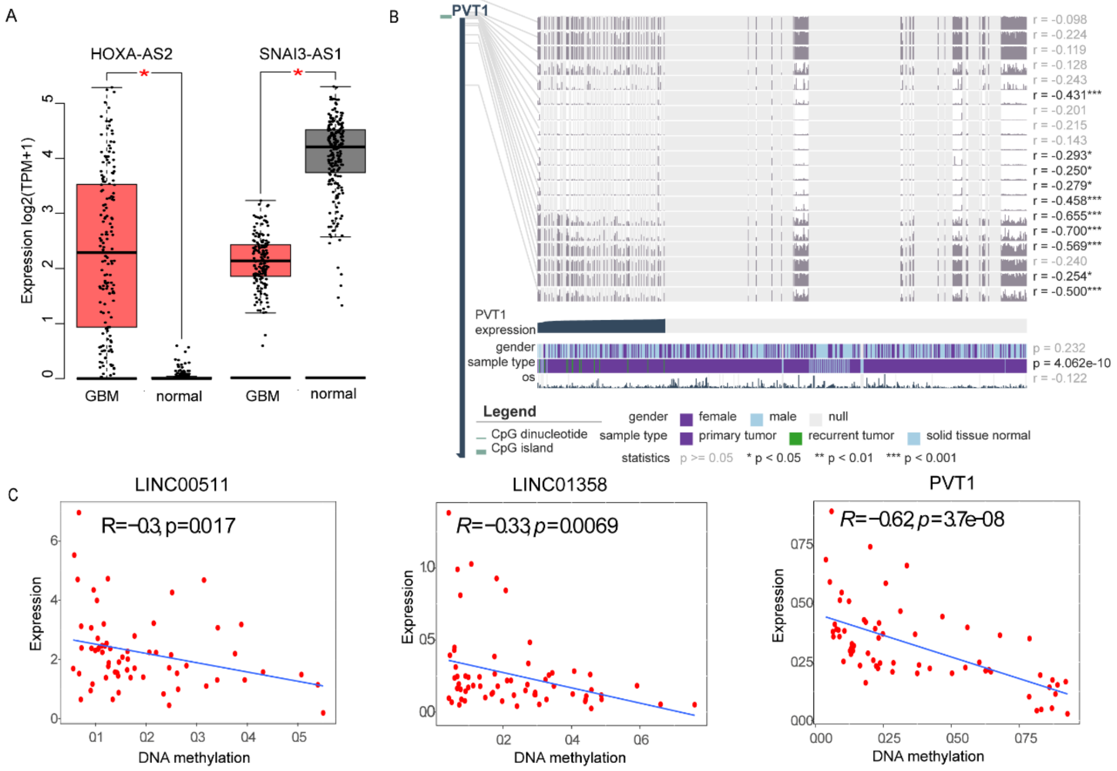

Figure S1. Recognition of differentially expressed and abnormally methylated lncRNAs.

(A) Gene expression boxplots of two differentially expressed lncRNAs. The expression of lncRNAs between GBM tumors and normal tissues. **(B)** The relationship between PVT1 methylation and its expression using MEXPRESS. Distribution of differentially methylated sites in PVT1 CpG island region. PVT1 methylation was inversely correlated with gene expression. **(C)** The Pearson correlation coefficients and corresponding P-values between lncRNA expression and DNA methylation level (β value).

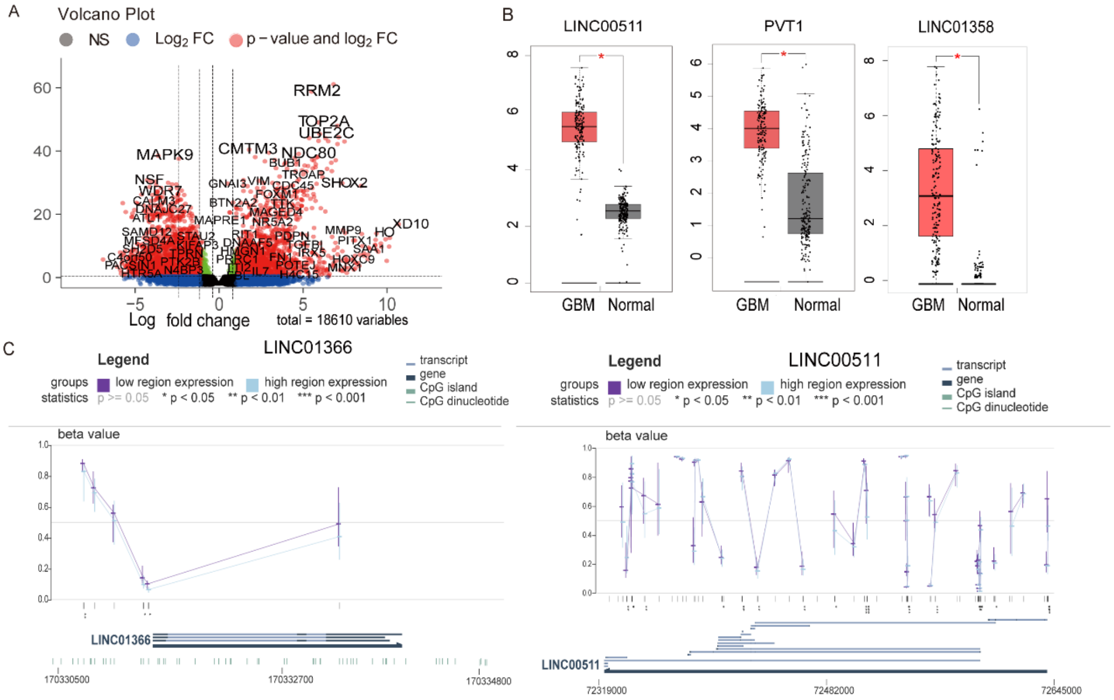

Figure S2. Identifying autophagy-related lncRNAs.

(A) Volcano plot showing up- and down-regulated genes in GBM. The differentially expressed gene of glioma is obtained by DEseq2 package (FDR<0.05). **(B)** Gene expression boxplots of three autophagy-related lncRNAs. The three autophagy-related lncRNAs are all differentially expressed lncRNAs in GBM. **(C)** The differentially methylated sites at the CPG positions of LINC01366 and LINC00511 were analyzed using MEXPRESS.

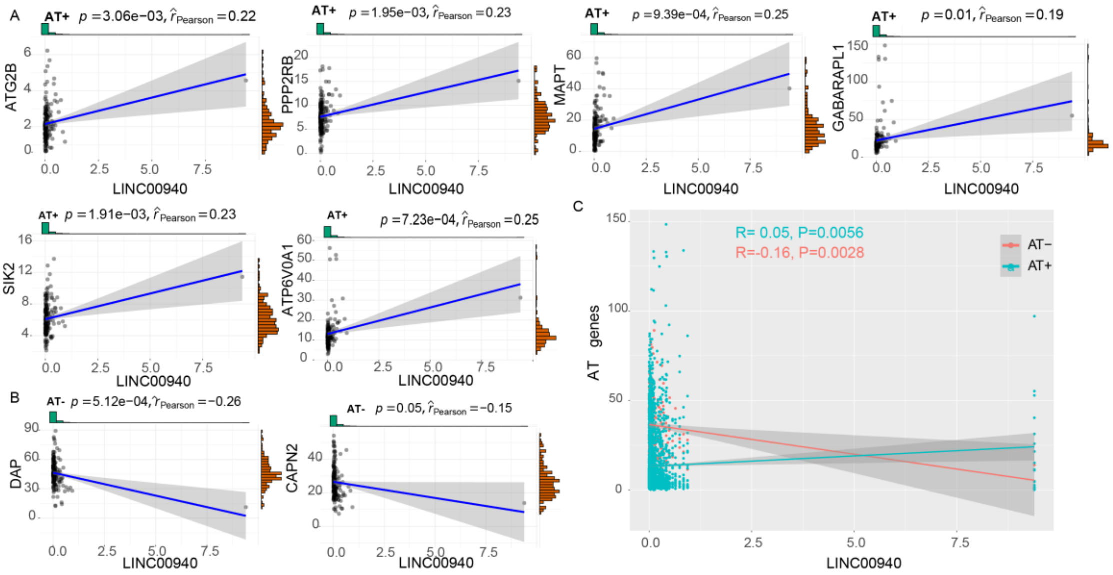

Figure S3. The correlation analysis of LINC00940 expression and autophagy-promoting or autophagy-inhibiting genes.

Pearson correlation coefficients for LINC00940 and autophagy-promoting genes (A)

and autophagy-inhibiting genes (B). (C) The distribution of Pearson correlation coefficients between LINC00940 and autophagy-promoting genes (blue; P=5.6e-3) and genes inhibiting autophagy (red; P=2.8e-3).

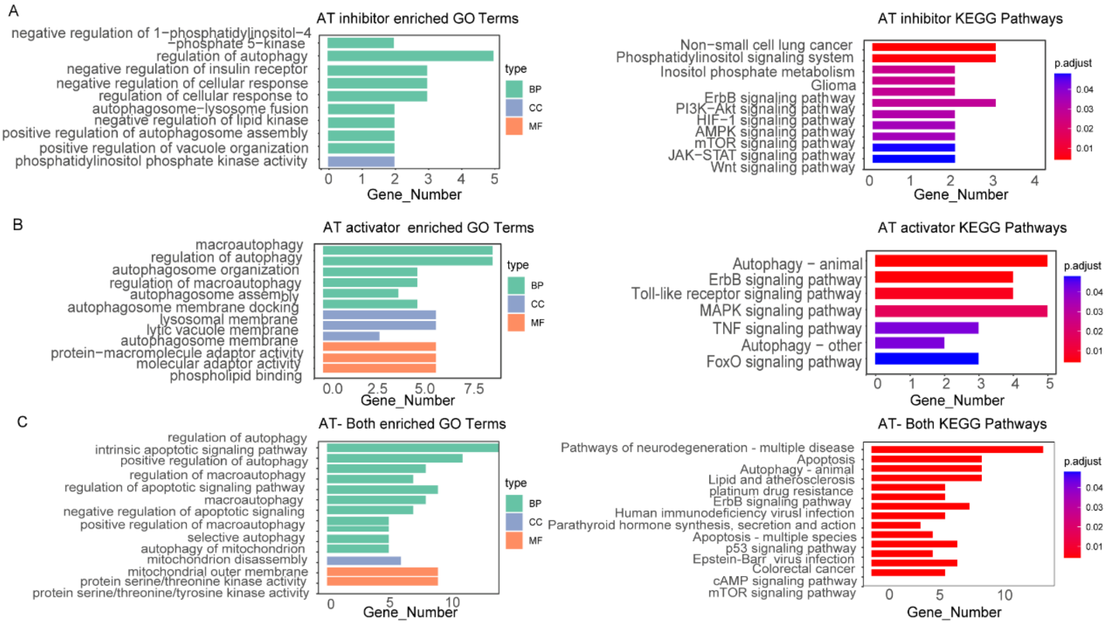

Figure S4. GO and KEGG functional enrichment of autophagy genes.

(A-C) GO enrichment and Kyoto Encyclopedia of Genes and Genomes (KEGG) pathway enrichment of the activitors, inhibitors and both activation and suppression of autophagy-related genes, respectively. Significant KEGG pathways and GO terms were obtained according to FDR <0.05.

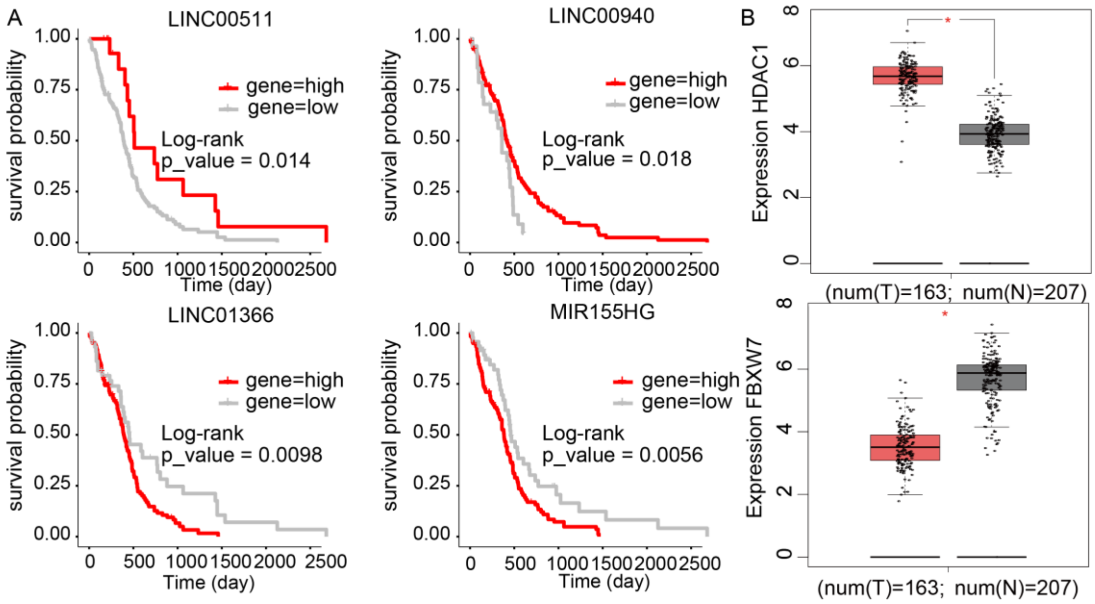

Figure S5. Analysis of survival-related genes.

(A) Comparison of differential expression of autophagy-related two target genes (HDAC1 and FBXW7) in glioma. Red indicates tumor samples, and gray indicates normal samples. **(B)** K-M OS curves based on the expression levels of LINC00511, LINC00940, LINC01366 and MIR155HG in patients with GBM in the TCGA dataset. The results of the log-rank test showed that these four lncRNAs were significantly

associated with GBM survival (P<0.05).

Table S1. Differentially expressed lncRNAs with aberrant methylation

FLJ12825	MIR155HG	LINC02488	LINC00513	LINC00457
LINC00298	PVT1	LINC01847	C8orf86	LINC02321
LINC00466	SALRNA1	DLEU2L	MIR124-2HG	LINC02346
LINC00504	LINC01965	LINC02525	LINC01151	LINC02192
LINC00511	MRPS9-AS2	LY86-AS1	TMEM72-AS1	TMEM220-AS1
LINC00940	LINC02084	LINC02828	PAUPAR	LINC01254
LINC01010	LINC00870	HOXA-AS2	MIR9-1HG	LINC00310
LINC01173	LINC00882	HOXA-AS3	APOA1-AS	LINC02817
LINC01358	LINC02016	HOXA10-AS	LINC02371	MIR3681HG
LINC01366	C1QTNF7-AS1	EGFR-AS1	LINC02617	SLC8A1-AS1
LINC01483	LINC00989	ELDR	LINC02389	EPCAM-DT
LINC01484	PRDM16-DT	LINC02577	RMST	

Table S2. The 9 autophagy-associated lncRNA regulators in GBM

lncRNA	P adj	ES	NES	Methylation	Region
LINC00511	0.0252	-0.292	-1.196	**hypomethylation**	**Promoter**
LINC00940	0.0170	0.589	1.276	**hypermethylation**	**Promoter**
LINC01010	0.0010	0.564	1.471	**hypermethylation**	**Promoter**
LINC01358	0.0025	-0.331	-1.394	**hypomethylation**	**Promoter**
LINC01366	0.0080	-0.298	-1.296	**hypomethylation**	**Promoter**
MIR155HG	0.0303	-0.345	-1.536	**hypomethylation**	**Promoter**
PVT1	0.0029	-0.363	-1.502	**hypomethylation**	**Promoter**
LINC00513	0.0079	0.247	0.558	**hypomethylation**	**Enhancer**
LINC01965	0.0020	0.222	0.540	**hypomethylation**	**Enhancer**

Table S3. Drug-lncRNA pairs

Drugs	lncRNA	Drugs	lncRNAs
Prestwick-984	LINC01358	guanethidine	LINC01010
pyrvinium	PVT1	iohexol	LINC01358
scriptaid	LINC00940	lactobionic acid	LINC01358
suramin sodium	LINC01010	lactobionic acid	LINC00511
syrosingopine	LINC01010	lovastatin	LINC01358
tolbutamide	LINC01010	midecamycin	LINC01010
trichostatin A	LINC01010	naringenin	LINC01358
trichostatin A	LINC00940	niclosamide	LINC01010
valproic acid	LINC00940	noscapine	LINC01010
vinburnine	LINC01010	oleandomycin	LINC01010
vorinostat	LINC00940	oxyphenbutazone	LINC00511
benzathine benzylpenicillin	LINC00940	rotenone	LINC00513
benzbromarone	LINC01358	sirolimus	LINC01965

2-aminobenzenesulfonamide	LINC01358	ursodeoxycholic acid	LINC01965
carisoprodol	LINC01358	cefalexin	LINC01965
cefadroxil	LINC01358	chlorhexidine	LINC00513
Chicago Sky Blue 6B	LINC01358	cimetidine	LINC00513
danazol	LINC00511	colecalciferol	LINC01965
diphemanil metilsulfate	LINC01010	convolamine	LINC01965
dobutamine	LINC01010	CP-944629	LINC00513
ethionamide	LINC01358	dioxybenzone	LINC00513
pepstatin	LINC00513	metoclopramide	LINC00513
		nalidixic acid	LINC00513

Table S4. The regulatory relationship between LINC00940 and drugs

Drug	ID	NES	P adj	PL
scriptaid	LINC00940	1.816	0.002	2
valproic acid	LINC00940	1.808	0.003	2
vorinostat	LINC00940	2.008	0.0004	2

Table S5. Functional enrichment results of MIR55HG

ID	Description	P value	Gene ID	Count
GO:0016236	Macroautophagy	2.08E-05	AP4M1/STAM/UBQLN2/VMP1	4
GO:0000045	Autophagosome assembly	2.86E-05	AP4M1/UBQLN2/VMP1	3
GO:1905037	autophagosome organization	3.19E-05	AP4M1/UBQLN2/VMP1	3

References

1. Xu C, Sun D, Wei C, Chang H. Bioinformatic analysis and experimental validation identified DNA methylation-Related biomarkers and immune-cell infiltration of atherosclerosis. *Front Genet.* 2022;13:989459.
2. Casas-Recasens S, Noell G, Mendoza N, et al. Lung DNA Methylation in Chronic Obstructive Pulmonary Disease: Relationship with Smoking Status and Airflow Limitation Severity. *Am J Respir Crit Care Med.* 2021;203(1):129-134.
3. Corces MR, Granja JM, Shams S, et al. The chromatin accessibility landscape of primary human cancers. *Science.* 2018;362(6413).
4. Shigeyasu K, Toden S, Ozawa T, et al. The PVT1 lncRNA is a novel epigenetic enhancer of MYC, and a promising risk-stratification biomarker in colorectal cancer. *Mol Cancer.* 2020;19(1):155.
5. Zhao H, Liu X, Yu L, et al. Comprehensive landscape of epigenetic-dysregulated lncRNAs reveals a profound role of enhancers in carcinogenesis in BC subtypes. *Mol Ther Nucleic Acids.* 2021;23:667-681.
